# Polymeric Pathogen-Like Particles-Based Combination Adjuvants Elicit Potent Mucosal T Cell Immunity to Influenza A Virus

**DOI:** 10.3389/fimmu.2020.559382

**Published:** 2021-03-04

**Authors:** Brock Kingstad-Bakke, Randall Toy, Woojong Lee, Pallab Pradhan, Gabriela Vogel, Chandranaik B. Marinaik, Autumn Larsen, Daisy Gates, Tracy Luu, Bhawana Pandey, Yoshihoro Kawaoka, Krishnendu Roy, M. Suresh

**Affiliations:** ^1^ Department of Pathobiological Sciences, University of Wisconsin-Madison, Madison, WI, United States; ^2^ The Wallace H. Coulter Department of Biomedical Engineering Georgia Institute of Technology and Emory University, Atlanta, GA, United States

**Keywords:** adjuvants, CD8, tissue-resident memory, CD4, influenza A virus, vaccine, polyfunctional

## Abstract

Eliciting durable and protective T cell-mediated immunity in the respiratory mucosa remains a significant challenge. Polylactic-co-glycolic acid (PLGA)-based cationic pathogen-like particles (PLPs) loaded with TLR agonists mimic biophysical properties of microbes and hence, simulate pathogen-pattern recognition receptor interactions to safely and effectively stimulate innate immune responses. We generated micro particle PLPs loaded with TLR4 (glucopyranosyl lipid adjuvant, GLA) or TLR9 (CpG) agonists, and formulated them with and without a mucosal delivery enhancing carbomer-based nanoemulsion adjuvant (ADJ). These adjuvants delivered intranasally to mice elicited high numbers of influenza nucleoprotein (NP)-specific CD8+ and CD4+ effector and tissue-resident memory T cells (T_RM_s) in lungs and airways. PLPs delivering TLR4 versus TLR9 agonists drove phenotypically and functionally distinct populations of effector and memory T cells. While PLPs loaded with CpG or GLA provided immunity, combining the adjuvanticity of PLP-GLA and ADJ markedly enhanced the development of airway and lung T_RM_s and CD4 and CD8 T cell-dependent immunity to influenza virus. Further, balanced CD8 (Tc1/Tc17) and CD4 (Th1/Th17) recall responses were linked to effective influenza virus control. These studies provide mechanistic insights into vaccine-induced pulmonary T cell immunity and pave the way for the development of a universal influenza and SARS-CoV-2 vaccines.

## Introduction

Respiratory infections in adults and children have been among the top three leading causes of death and disability in the world for decades (1, 2). Novel respiratory pathogens are emerging and can quickly spread due to the ease of transmission, as witnessed in the current coronavirus (3–5) and past influenza pandemics (6). Vaccines are essential for the control and elimination of these diseases by eliciting antibody and/or T cell-mediated immune responses (CMI). The necessity for effective T cell-based vaccines to respiratory pathogens is exemplified by continued seasonal influenza endemics and sporadic pandemics, despite wide vaccine availability and high virus infection rates.

Widely used inactivated influenza vaccines (IIV) function by eliciting subtype-specific antibodies to mutation-prone surface hemagglutinin (HA) and neuraminidase (NA) proteins, and must be reformulated annually to match seasonal strains due to antigenic drift and shift (7). Further, these IIVs are poorly immunogenic for CD8 T cells. Influenza viral infection can generate strong antibody and T cell-mediated immunity (CMI), and while antibodies are still strongly strain matched in terms of protection, heterosubtypic protection to influenza requires CMI to conserved viral epitopes (7, 8).

Robust T cell control of heterosubtypic influenza infection has been closely associated with the development of mucosally residing tissue resident memory (T_RM_) CD4+ and CD8+ cells (9, 10). However, the numbers of T_RM_ cells after influenza infection have been observed to wane over time with a concomitant decrease in heterosubtypic protection (10–12). Indeed, neither mass vaccination with current IIV or widespread infection have adequately curtailed the continuous infection of influenza virus in human populations. Therefore, development of vaccination strategies to potently elicit long-lived T_RM_ cells and understanding of what factors govern these responses are crucially needed to control respiratory infections such as influenza.

Several groups have developed vaccines to elicit influenza-specific lung T_RM_ cells that confer heterosubtypic protection to influenza virus challenge using live attenuated influenza viruses (13), or viral vectors (14). Recently, we have tested a novel adjuvant, Adjuplex (ADJ, a nano emulsion of carbomer and soy lethicin (15)), that when combined with subunit or IIV proteins potently induced CD8+ lung T_RM_ cell responses after mucosal administration, and conferred substantial protection to influenza virus challenge (16, 17). These studies highlight the importance for identification of novel adjuvants that can elicit mucosal CMI to non-replicating antigens, particularly so we can dissect and study the individual effects these adjuvants have on the magnitude and nature of the resultant mucosal CMI responses.

TLR ligands as adjuvants for influenza vaccines have been widely studied and are typically delivered in monomeric, soluble formulations (18). In order to mimic biophysical interactions between pattern-recognition receptors and their ligands on pathogens, we have developed TLR agonist-loaded polylactic-co-glycolic acid (PLGA)-based pathogen-like particles (PLPs) (19). Varying the size of the PLPs and the density of the loaded TLR agonist CpG, modulated the signaling circuitry within dendritic cells *in vitro* and altered the nature of antibody (T_H_1 versus T_H_2-driven) responses. Additionally, agonists presented simultaneously on PLPs have been shown to differentially modulate immune responses *in vitro*, compared with soluble counterparts, while potentially improving safety by reducing toxicity from systemic diffusion (20, 21). The ability of TLR-agonist-loaded PLPs to stimulate antigen-specific T cell responses, especially in the respiratory mucosa, has not yet been investigated.

Here, we investigated whether CpG- or glucopyranosyl lipid adjuvant (22) (GLA)-loaded PLP adjuvants mixed with influenza virus nucleoprotein (NP) protein, with or without ADJ could elicit antigen-specific CD4 and CD8 T cells, and analyzed their numbers, frequencies, and phenotypes in mucosal compartments. We found that PLP vaccine formulations elicited strong, yet phenotypically and functionally distinct T_RM_ CD8+ and CD4+ responses in the lungs of vaccinated mice. Further, we observed that PLP-based adjuvants afforded strong and durable protection from influenza challenge that was closely associated with distinct functional recall profiles of CD4 and CD8 T cells unique to the PLP vaccine formulation. These results highlight that PLP-loaded adjuvants can distinctly program effective CMI and can be leveraged to study immunity and develop vaccines to respiratory pathogens such as influenza virus and SARS-CoV-2.

## Methods

### Experimental Animals

Six- to 12-week-old C57BL/6J (B6) were purchased from Jackson Laboratory or from restricted-access specific-pathogen-free (SPF) mouse breeding colonies at the University of Wisconsin-Madison Biotron Laboratory. All mice were housed in SPF conditions in the animal facilities at the University of Wisconsin-Madison (Madison, WI).

### Ethics Statement

These studies were carried out in strict accordance with recommendations set forth in the National Institutes of Health Guide for the Care and Use of Laboratory Animals. All animals and animal facilities were under the control of the School of Veterinary Medicine with oversight from the University of Wisconsin Research Animal Resource Center. The protocol was approved by the University of Wisconsin Animal Care and Use Committee (Protocol number V005308). The animal committee mandates that institutions and individuals using animals for research, teaching, and/or testing much acknowledge and accept both legal and ethical responsibility for the animals under their care, as specified in the Animal Welfare Act (AWA) and associated Animal Welfare Regulations (AWRs) and Public Health Service (PHS) Policy.

### Cells and Viruses

Murine bone marrow-derived dendritic cells (BMDCs) were differentiated from bone marrow cells isolated from C57BL/6 mice. The bone marrow was processed into single cell suspensions and treated with RBC lysis buffer. Cells were then plated into Petri dishes and cultured in BMDC differentiation medium (RPMI, 10% FBS, 1% penicillin-streptomycin, 1x sodium pyruvate, 1x β-mercaptoethanol) supplemented with GM-CSF (PeproTech, Rocky Hill, NJ). Media was refreshed on days 2, 4, and 6. On day 7, BMDCs were harvested and replated for experiments.

Madin-Darby canine kidney (MDCK) cells were obtained from ATCC (ATCC; Manassas, VA, USA) and propagated in growth media containing Modified Eagle’s Medium (MEM) with 10% fetal bovine serum (FBS; Hyclone, Logan, UT), 2 mM L-glutamine, 1.5 g/l sodium bicarbonate, non-essential amino acids, 100 U/ml of penicillin, 100 μg/ml of streptomycin (flu media), and incubated at 37°C in 5% CO2. Reverse genetics-derived influenza virus strain A/PR/8/34 H1N1 (PR8) were propagated in MDCK cells, and viral titers were determined by plaque-forming assay (23, 24). Briefly, MDCK cells grown to 90% confluency were infected with serial dilutions of influenza virus samples, and incubated for 1 h while periodically shaking under growth conditions. Cells were then washed with PBS and incubated in flu media containing 1% SeaPlaque agarose (Lonza, Basel, Switzerland). After 48 h incubation, cells were fixed in 10% neutral buffered formalin (NBF), agarose plugs were removed, and distinct plaques were counted at a given dilution to determine the plaque forming units (PFU) of virus per sample.

### Viral Challenge

For PR8 challenge studies, mice were inoculated with 500 PFU of PR8 by intranasal (IN) instillation in 50ul PBS applied to the nares under isoflurane anesthesia, and were humanely euthanized at 6 days post infection. Lung tissues for viral titration (left lobe) were frozen at −80°C. To assess the role of CD4 T cells and CD8 T cells in protective immunity, mice were administered 200 μg of anti-CD4 (Bio X Cell, Clone: GK1.5) or CD8 T cells (Bio X Cell; Clone 2.43) intravenously and intranasally at days -5. -3, -1 and 1, 3, and 5 relative to challenge with influenza A virus. Fingolimod (FTY720, Selleck Chemicals) was administered to mice by intravenous injection at a dose of 5mg/kg bodyweight on days. -3, -1 and 1, 3, and 5 relative to challenge with influenza virus.

### Vaccines and Vaccinations

PR8 nucleoprotein (NP) was purchased from Sino Biological Inc (Beijing, China). CpG ODN 1826 (CpG) oligonucleotide adjuvant was purchased from InivivoGen (San Diego, CA). The synthetic monophosphoryl lipid A adjuvant, Glucopyranosyl Lipid Adjuvant (GLA) was purchased from Avanti Polar Lipids, Inc. (Alabaster, AL). Adjuplex is a proprietary preparation consisting of an emulsion of polyacrylic acid and soy lecithin, purchased from Advanced BioAdjuvants, LLC. PLPs were synthesized by the double emulsion method. Briefly, PLGA was dissolved in dichloromethane in the presence or absence of GLA adjuvant (10 μg GLA/mg PLGA). DI H_2_O was added and the mixture was homogenized to create the first emulsion. One percent PVA was then added and the mixture was homogenized to create the second emulsion. Excess dichloromethane was removed by solvent evaporation and particles were washed with DI H_2_O by centrifugation. Following lyophilization, branched PEI was conjugated to the PLP surface by reaction with EDC and sulfo-NHS (Thermo Fisher Scientific, Bedford, MA). PLPs were washed again sequentially with 1 mM NaCl and DI H_2_O. CpG adjuvant was loaded onto PLPs (10 μg CpG/mg PLGA) without GLA in sodium phosphate buffer (pH = 6.5) overnight at 4C. Particle size and zeta potential at pH 7.4 was measured using a Malvern Zetasizer and were measured to be within range of what was previously reported for both PLP-CpG and PLP-GLA/MPLA particles (PLP-MPLA/GLA 1.69 μm ± 0.29 μm, -7.59 mV ± 0.15 mV; PLP-CpG 1.72 μm ± 0.37 μm, -20.56 mV ± 4.62 mV) (21). All vaccinations were given *via* IN instillation under isoflurane anesthesia in 50μl saline with 10 μg NP formulated in various adjuvants as follows: 10% ADJ (ADJ) +/-; 1 mg PLGA (PLP-E); 1 mg PLGA loaded with 10μg CpG (PLP-CpG); 1 mg PLGA loaded with 10 μg GLA (PLP-GLA); 10% ADJ. For all studies, mice were boosted with an identical dose 3 weeks after primary vaccination.

### BMDC Activation and Proliferation

Murine BMDCs were plated in 96-well plates (300,000 cells/well). BMDCs were incubated with ADJ (1%) and/or PLP adjuvants (50 μg PLGA/mL). After 24 h, supernatants were collected. IFN-β, IL-1β, and IL-18, were measured by ELISA (Bio-Techne, Minneapolis, MN). Cells were then incubated with CellTiter 96 Aqueous One Solution Proliferation Solution for 1 h (Promega, Fitchburg, WI). Absorbance of the solution was then read at 490 nm. Measurements were normalized to untreated cells at the same timepoint of incubation.

### Flow Cytometry

For indicated studies, vascular staining of T-cells was performed by IV injection of fluorochrome-labeled CD45.2 3 min prior to animal euthanasia. Single-cell suspensions from spleen and lung were prepared using standard techniques as described (17). Bronchoalveolar lavage (BAL) cells were collected from euthanized mice by cannulating the trachea and flushing 3 times with 1 ml cold 10% FBS-RPMI, followed by cell pelleting. Prior to antibody staining, cells were stained for viability with Fixable Viability 780 (eBioscience, San Diego, CA) according to manufacturer’s instructions. Fluorochrome-labeled antibodies against the cell-surface antigens, Ly5.2 (CD45.2), CD4, CD8α, CD44, CD62L, KLRG-1, CD127, CD103, CD69, CD49A, CD127, CXCR3, CX3CR1, and intracellular antigens IFN-γ, TNF-α, IL-2, IL-17, TBET, EOMES, IRF-4, and granzyme B were purchased from BD Biosciences (San Jose, CA), BioLegend (San Diego, CA), eBioscience (San Diego, CA), Invitrogen (Grand Island, NY), or Tonbo Biosciences (**Supplementary Table 2**). Fluorochrome-conjugated I-A^b^ and H-2/D^b^ tetramers bearing influenza nucleoprotein peptides, QVYSLIRPNENPAHK (NP311) and ASNENMETM (NP366), respectively, were kindly provided by the NIH Tetramer Core Facility (Emory University, Atlanta, GA). For class-II tetramer NP311, cells were incubated at 37°C for 90 min. For class-I tetramers, cells were incubated with tetramer and antibodies for 60 min on ice in the dark. Stained cells were fixed with 2% paraformaldehyde in PBS for 20 min, then transferred to FACS buffer. All samples were acquired on a LSRFortessa (BD Biosciences) analytical flow cytometer. Data were analyzed with FlowJo software (TreeStar, Ashland, OR).

### Intracellular Cytokine Stimulation

For intracellular cytokine staining, one million  cells were plated on flat-bottom tissue-culture-treated 96-well plates. Cells were stimulated for 5 h at 37°C in the presence of human recombinant IL-2 (10 U/well), and brefeldin A (1 μl/ml, GolgiPlug, BD Biosciences), with one of the following peptides: NP366, NP311 (thinkpeptides^®^, ProImmune Ltd. Oxford, UK) at 0.1 ug/ml, or without peptide. After stimulation, cells were stained for surface markers, and then processed with Cytofix/Cytoperm kit (BD Biosciences, Franklin Lakes, NJ). Figures presenting cytokine expression in this manuscript are from peptide stimulated cells only, as unstimulated cells produced low frequencies and levels of cytokines determined by intracellular staining (**Supplementary Figure 8**)

### Statistical Analyses

Total cell numbers are calculated = frequency (percent/100) of marker expression in live cells **x** total cell count per tissue. Statistical analyses were performed using GraphPad software (La Jolla, CA). All comparisons were made using one-way ANOVA test with Tukey corrected multiple comparisons where p<0.05 = *, p<0.005 = **, p<0.0005 = ***, etc. were considered significantly different among groups. Viral titers were log transformed prior to analysis. Error bars in all figures represent the standard error of the mean (SEM). For correlation analysis, a simple linear regression was performed and significance values represent if the slope was significantly non-zero.

## Results

### Adjuplex Modifies Responses of Murine BMDCs to TLR Agonist-Loaded PLPs

**Figure 1:**
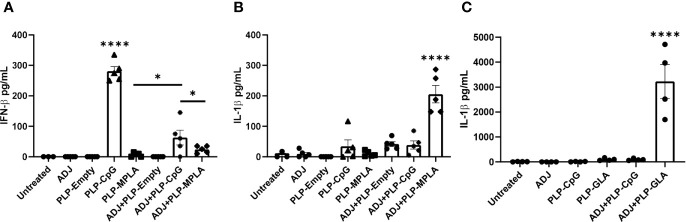
Murine BMDC response to Adjuplex and TLR agonist-loaded PLP adjuvants. Murine BMDCs were treated with Adjuplex (ADJ) and/or PLPs with CpG, MPLA or GLA. **(A)** IFN-β response from BMDCs after 24 h of treatment with ADJ and/or PLP-CpG or PLP-MPLA. **(B, C)** IL-1β response from BMDCs after 24 h of treatment with ADJ and/or PLP-CpG, PLP-MPLA or PLP-GLA. Data are representative of two independent experiments. *, and **** indicate significance at P<0.1 and 0.0001 respectively.

We first assessed the extent to which ADJ modulated the responses of murine DCs to TLR agonists CpG and monophosphoryl lipid A (MPLA) presented as PLPs. Data in **Figure 1A** show that PLP-CpG triggered potent IFN-β responses from murine BMDCs. Interestingly, ADJ or PLP-MPLA alone did not induce an IFN-β response, but ADJ dampened the IFN-β response induced by PLP-CpG (**Figure 1A**). Next, we explored whether MPLA and synthetic MPLA (GLA) triggered production of other pro-inflammatory cytokines such as IL-1β in BMDCs. Data in **Figures 1B, C** show that ADJ, PLP-CpG, PLP-MPLA, or PLP-GLA alone failed to induce IL-1β from murine BMDCs. However, ADJ+PLP-MPLA and ADJ+PLP-GLA but not ADJ+CpG induced strong IL-1β response in DCs (**Figures 1B, C** **)**. Taken together, data in **Figure 1** showed that ADJ in combination with CpG and MPLA/GLA stimulated disparate cytokine responses from BMDCs.

### Combination PLP Adjuvants Elicit Contrasting CD8 and CD4 T Cell Effector Responses

**Figure 2:**
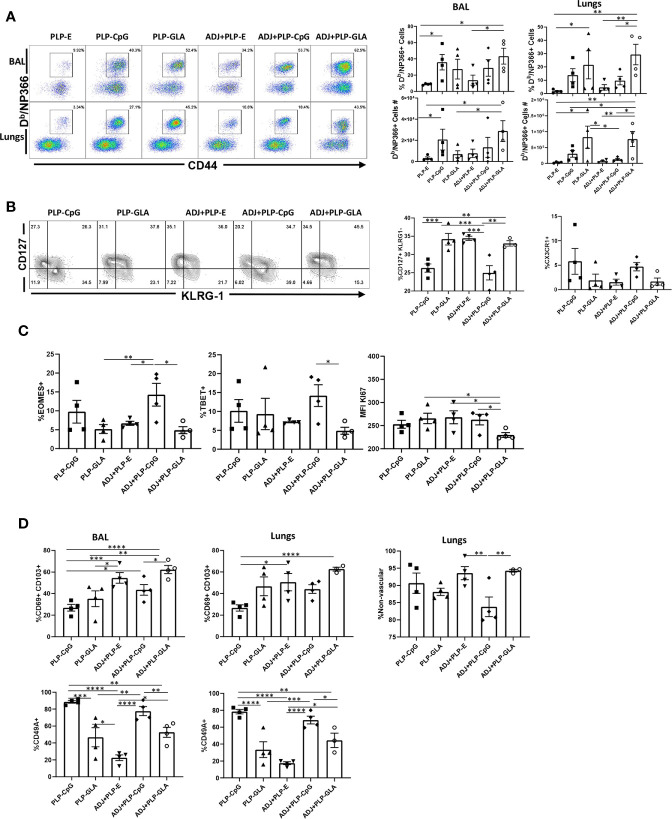
Effector CD8 T cell response to adjuvanted vaccines. C57BL/6J mice were vaccinated intranasally twice (3 weeks apart) with influenza virus nucleoprotein (NP) formulated with the indicated adjuvants. At day 8 post boost, cells in the lungs and bronco-alveolar lavage (BAL) were stained with D^b^/NP366 tetramers and the indicated antibodies. **(A)** FACS plots are gated on live CD8 T cells and the numbers are the percentages of tetramer-binding CD8 T cells among CD8 T cells. FACS plots and graphs in panels **(B–D)** show indicated percentages of the subsets among gated tetramer-binding CD8 T cells in respective gates/quadrants or median fluorescence intensities (MFI) for the molecules as indicated. Data are representative of three independent experiments. *, **, ***, and **** indicate significance at P < 0.1, 0.01, 0.001, and 0.0001, respectively.

Here, we compared the ability of various TLR agonist-loaded PLPs formulated with or without ADJ to elicit pulmonary CD8 T cell responses to the subunit protein, influenza virus nucleoprotein (NP). Mice were vaccinated intranasally (IN) twice at an interval of 3 weeks. On the 8^th^ day after the booster vaccination, we quantified NP-specific effector T cell responses in lungs and airways. Gating strategy for visualizing NP-specific CD8 T cell responses is shown (**Supplementary Figure 1**). All PLP adjuvants elicited robust CD8 T cell responses in the lungs and airways of vaccinated mice (**Figure 2A**). Remarkably a mean of 30%–50% of CD8 T cells in the airways (BAL) were specific to the immunodominant epitope NP366 in PLP-CpG and PLP-GLA groups (**Figure 2A**). Combination of PLP-CpG or PLP-GLA with ADJ did not significantly (P<0.05) affect the frequencies of such cells in lungs or airways. While PLP-GLA+/-ADJ seemed to drive the highest numbers of NP366-specific CD8 T cells in lungs, PLP-CpG groups had lower levels of these effector cells. Mice administered NP with empty PLP (PLP-E) had relatively low frequencies and numbers of NP366-specific CD8 T cells, indicating that the PLP particles alone did not substantively contribute to the induction of this immune response, and were excluded from further phenotypic analysis due to unreliably low numbers (**Supplementary Figure 1B**).

We assessed whether adjuvants differed in terms of regulating the differentiation of effector T cells. T cells expressing higher levels of IL-7 receptor (CD127) and lower levels of a terminal differentiation/senescence marker KLRG-1 are associated with greater memory potential (25). Of the PLP preparations tested, PLP-CpG and ADJ+PLP-CpG induced significantly (P<0.05) lower frequencies of CD127^HI^ and CD127^HI^/KLRG-1^LO^ CD8 T cells than all other combination PLP adjuvants (**Figure 2B** and **Supplementary Figure 2**). PLP-CpG also drove a strong trend for increased frequencies of CX3CR1^HI^ CD8 T cells (**Figure 2B**), a marker associated with increased effector differentiation (26). We quantified transcription factors EOMES and TBET, which are known to regulate differentiation of effector CD8 T cells in spleen (27). Interestingly, while the combination of ADJ+PLP-CpG induced high levels of transcription factors TBET and EOMES, the combination of ADJ+PLP-GLA had a suppressive effect, leading to lower levels of these transcription factors and lower levels of KI67 expression, a marker of cell proliferation (**Figure 2C**). Thus, increased expressions of EOMES and TBET were associated with greater terminal differentiation of CD8 T cells in the ADJ+PLP-CpG group.

To determine whether combination PLP adjuvants differentially regulated mucosal imprinting of lung CD8 T cells, we analyzed CD69 and CD103 expression. ADJ and PLP-GLA both increased CD69^HI^/CD103^HI^ CD8 T cells in the lungs of vaccinated animals, while PLP-CpG appeared to decrease this mucosal imprinting (**Figure 2D**). The combination of ADJ+PLP-GLA led to significantly (P<0.05) higher levels of mucosal imprinting in airways and lungs compared with PLP-CpG, ADJ+PLP-CpG, or PLP-GLA; ADJ alone strongly induced CD69 and CD103 expression. Another marker that is expressed on lung tissue-resident memory cells (T_RM_), is CD49a (28). In our studies, CD49A expression on CD8 T cells appeared to be closely associated with PLP-CpG treatment (**Figure 2D**), and unlike CD69 and CD103, CD49a was expressed to significantly lower levels on CD8 T cells from groups administered ADJ+/-PLP-GLA. To determine the localization of effector CD8 T cells in lungs, we performed vascular staining of T cells shortly before euthanasia. Levels of non-vascular cells were highest in the ADJ and ADJ+PLP-GLA groups, which more closely associated with CD69/CD103 levels than CD49A levels (**Figure 2D**). Additionally, we did not observe a difference in CD69/CD103 positivity between total and exclusively non-vascular NP366-specifc CD8 T cells (**Supplementary Figure 3**) because the vast majority of these cells, in all treatment groups, were found in the non-vascular compartment. Thus, ADJ+/-PLP-GLA enhanced expression of CD103 and CD69, and promoted mucosal imprinting of effector CD8 T cells in lungs. By contrast, PLP-CpG appeared to dampen the expression of CD103/CD69, and limit non-vascular localization of effector CD8 T cells.

**Figure 3:**
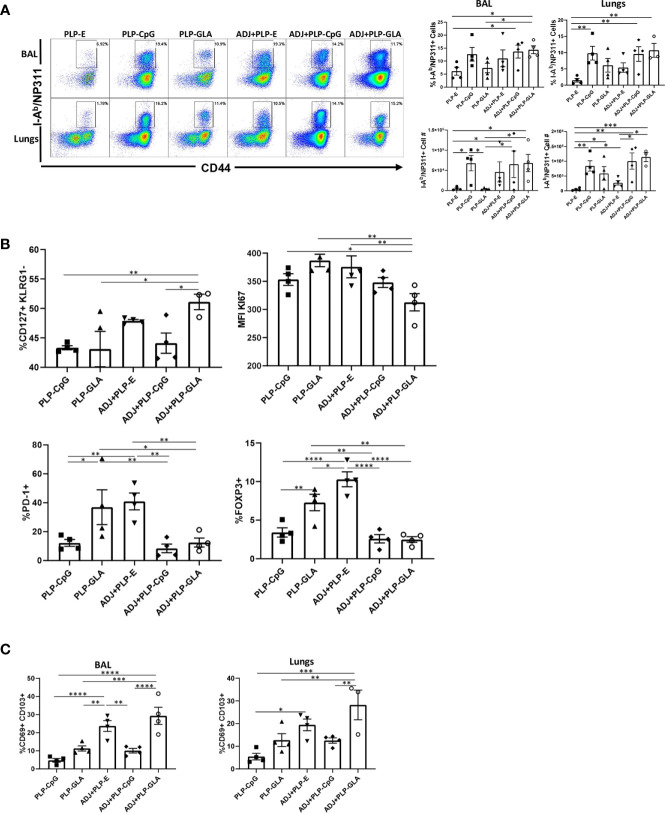
Effector CD4 T cell response to PLP-based vaccines. Cohorts of mice were vaccinated as in **Figure 2**. At day 8 post booster vaccination, cells from lungs and BAL were stained with I-A^b^/NP311 tetramers and the indicated antibodies. **(A)** FACS plots show numbers and percentages of tetramer-binding cells among CD4 T cells. Graphs in **(B, C)** show percentages of subsets among gated tetramer-binding CD4 T cells in respective gates/quadrants or median fluorescence intensities (MFI) for the indicated molecules. Data are representative of three independent experiments. *, **, ***, and **** indicate significance at P < 0.1, 0.01, 0.001, and 0.0001, respectively.

CpG/GLA-loaded PLPs+/-ADJ elicited high frequencies of antigen-specific CD4 T cells in lungs and airways (**Figure 3A**). GLA- and/or CpG-loaded PLP adjuvants induced higher accumulation of NP311-specific CD4 T cells in lungs compared to groups that received empty PLPs (**Figure 3A**). Similar to CD8 T cell responses, combination of ADJ+PLP-GLA showed a trend for highest levels of antigen-specific responses, however in contrast to NP366-specific CD8 T cell responses, PLP-CpG+/-ADJ also appeared sufficient to induce high frequencies and numbers of NP311-specific CD4 T cells in the respiratory tract (**Figure 3A**).

Pertaining to the differentiation of effector CD4 T cells, only the combination of ADJ+PLP-GLA induced greater percentages of CD127^HI^/KLRG-1^LO^ CD4 T cells, relative to other treatment groups (**Figure 3B**). The expression of CX3CR1 and transcription factors (TBET/EOMES/IRF4) were not significantly (P<0.05) different between treatment groups (**Supplementary Figure 4A**). However, CD4 T cells isolated from mice that were vaccinated with ADJ+PLP-GLA appeared to be proliferating at significantly (P<0.05) lower levels, as measured by lower Ki67 staining (**Figure 3B**). Interestingly, the frequencies of PD-1^HI^ NP311-specific CD4 T cells and FoxP3^HI^ regulatory CD4 T cells were significantly (P<0.05) higher in both the PLP-GLA and ADJ+PLP-E groups. Similar to the findings for CD8 T cells, ADJ and ADJ+PLP-GLA fostered mucosal imprinting of CD4 T cells, as these groups had significantly (P<0.05) higher frequencies of CD69^HI^/CD103^HI^ NP311-specific CD4 T cells (**Figure 3C**). However, CD49A expression and proportions of non-vascular T cells were not significantly different between treatment groups (**Supplementary Figure 4B**).

### Distinct Functional Programming of CD8 and CD4 T Cells by Combination PLP Adjuvants

**Figure 4:**
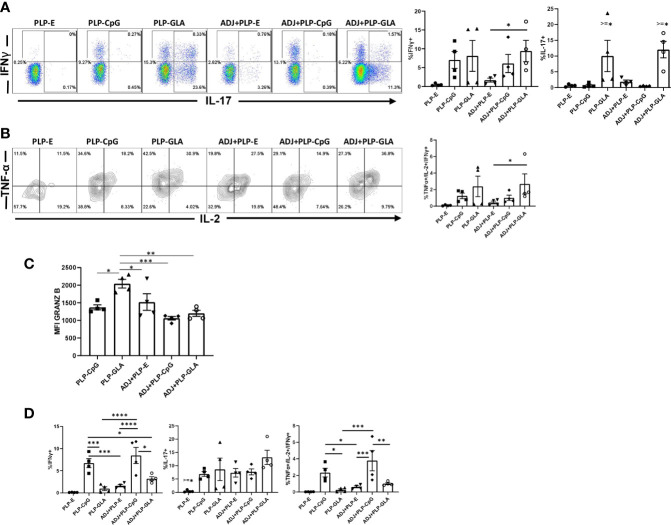
Functional polarization of effector CD8 and CD4 T cells in vaccinated mice. Mice were vaccinated as described in **Figure 2**. On the 8^th^ day after booster vaccination, lung cells were stimulated *ex vivo* with NP366 or NP311 peptides for 5 h. The percentages of NP366 peptide-stimulated CD8 T cells or NP311 peptide-stimulated CD4 T cells that produced IFN-γ, IL-17, TNF-α, and IL-2 were quantified by intracellular cytokine staining. **(A)** FACS plots and graphs show the percentages of cytokine-producing cells among the gated CD8 T cells. **(B)** Data in graphs are percentages among gated IFN-γ-producing CD8 T cells. **(C)** Graphs show the percentages of cytokine-producing cells among CD4 T cells or IFN-γ-producing CD4 T cells (TNF^+^IL-2^+^). **(D)** Cells were stained with anti-CD8, D^b^/NP366 tetramers and anti-granzyme B antibodies directly ex vivo. Graph shows MFI for granzyme B staining in NP366-specific CD8 T cells. Data are representative of three independent experiments. *, **, ***, and **** indicate significance at P < 0.1, 0.01, 0.001, and 0.0001, respectively.

At day 8 after booster vaccination, we assessed cytokine production by NP366 peptide-stimulated CD8 T cells in lungs. All PLP-based adjuvants elicited strong CD8 Tc1 responses, measured by IFNγ secretion after *ex vivo* NP366 peptide stimulation (**Figure 4A**), with the exception of the PLP-E which failed to elicit any detectable cytokine expression from stimulated cells. In general, the frequency of IFNγ-secreting CD8 T cells from PLP-CpG/PLP-GLA groups were at least two-folds higher than the ADJ+PLP-E group, which suggested that PLP-TLR4/9 agonists + ADJ favored Tc1 programming. Production of IL-17α in CD8 T cells was significantly increased by PLP-GLA-containing adjuvant formulations, yet was barely detectable in PLP-CpG groups, highlighting the contrasting functional programming of TLR4 and TLR9 agonists, respectively (**Figure 4A**). Though not statistically significant (P<0.05), there was a trend for higher polyfunctionality as measured by frequency of TNFα+/IL-2+/IFNγ+ CD8 T cells in PLP-GLA mice +/- ADJ (**Figure 4B**). Overall, PLP-CpG adjuvants appear to skew towards Tc1 polarization, while PLP-GLA resulted in balanced Tc1/Tc17 responses and greater functional diversity. We also evaluated whether adjuvants differed in terms of inducing effector differentiation, as measured by expression of granzyme B. Expression of the effector molecule granzyme B in CD8 T cells was not associated with administration of PLP-CpG, and conversely NP366-specific CD8 T cells in mice receiving PLP-GLA had significantly (P<0.05) higher levels for granzyme B, compared to all other adjuvant combinations (**Figure 4C**).

In stark contrast to CD8 T cell functionality, PLP-CpG tended to drive balanced Th1/Th17 responses in CD4 T cells, compared with PLP-GLA, which directed skewed T_H_17 differentiation (**Figure 4D**). PLP-CpG+/-ADJ groups had significantly (P<0.05) higher frequencies of IFNγ+ CD4 T cells, while all combination PLP adjuvant groups exhibited similar frequencies of IL-17-producing CD4 T cells (**Figure 4D**). Further, the PLP-CpG+/-ADJ groups had higher frequencies of polyfunctional triple cytokine-producing- (IFNγ, TNF, and IL-2) CD4 cells than the ADJ+PLP-E and PLP-GLA groups (**Figure 4D**). When the functional polarization of CD4 and CD8 T cells is considered together, ADJ+PLP-GLA induced the most balanced and potent Tc1/Tc17 and Th1/Th17 immunity. These results also demonstrated that the type of TLR agonist conjugated to PLPs can have disparate programming effects on CD4 and CD8 T cell functionality during the effector phase of vaccination.

### PLP Adjuvants Affect the Magnitude and Functionality of CD4 and CD8 T Cell Memory

**Figure 5:**
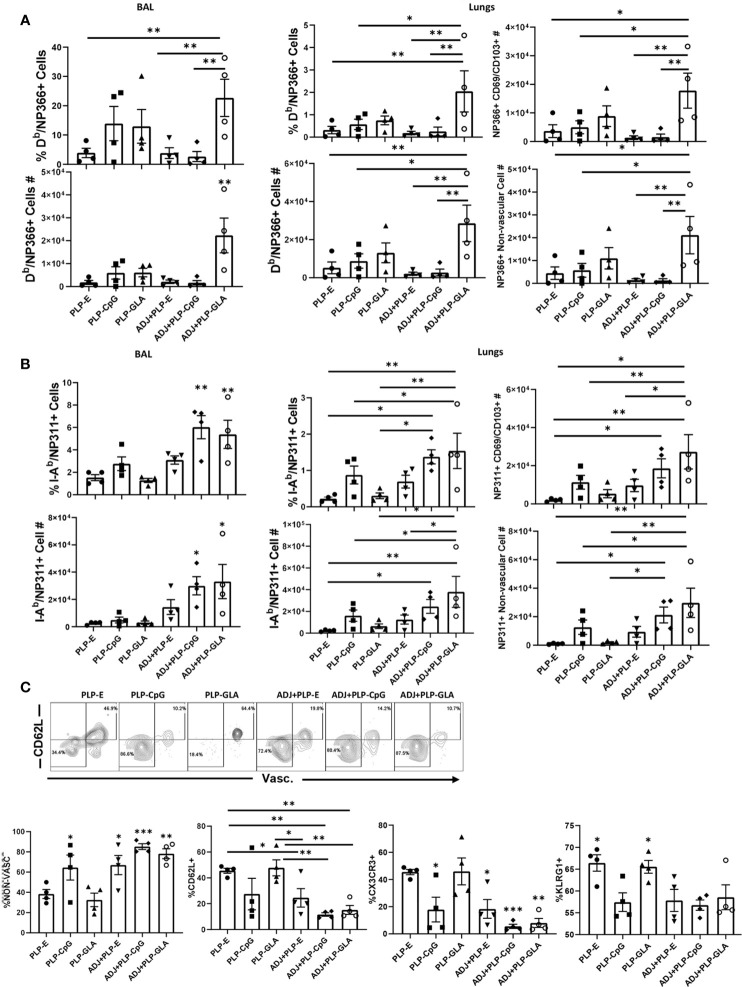
Mucosal CD8 and CD4 T cell memory in vaccinated mice. Cohorts of mice were vaccinated twice, as described in **Figure 2**. At 100 days after booster vaccination, to stain vascular cells, mice were injected intravenously with PE-labeled anti-CD4.2 antibody. Cells from BAL and lungs were stained with D^b^/NP366 tetramers or I-A^b^/NP311 tetramers along with antibodies for the indicated cell surface molecules. Percentages and total numbers of NP366-specific CD8 T cells or NP311-specific CD4 T cells in BAL or lungs (**A, B**, respectively). Graphs in panels **(A, B)** also show total numbers of CD103^+^CD69^+^ and nonvascular NP366- or NP311-specific T cells in lungs. Plots in **(C)** are gated on NP311-specific T cells and graphs show percentages of the indicated subsets among tetramer-binding CD4 T cells. Data are representative of three independent experiments. *, **, and *** indicate significance at *P*<0.1, 0.01, and 0.001, respectively.

Cohorts of mice were vaccinated twice (3 weeks apart) with NP protein formulated in various adjuvants, and NP-specific memory T cells were quantified in the lungs and airways, at 100 days after booster vaccination. While all combination PLP adjuvants elicited readily detectable levels of NP366-specific memory CD8 T cells at D100 post boost, the ADJ+PLP-GLA group had significantly (P<0.05) higher frequencies and/or total numbers of memory CD8 T cells in both airways and lungs than other groups (**Figure 5A**). The greater numbers of memory CD8 T cells in lungs of ADJ+PLP-GLA mice are linked to reduced contraction between day 8 and day 100 after vaccination; while the number NP366-specific CD8 T cells dropped by ~100 fold in other groups, there was only a ~20-fold drop in the ADJ+PLP-GLA group (**Supplementary Figure 5**). There were no substantial differences in CD49a, CD62L, CD69, CD103, CD127, CXCR3, CX3CR1, or KLRG1+ frequencies among NP366-specific memory CD8 T cells in various adjuvant groups. Notably however, the numbers of non-vascular parenchymal CD69^+ve^CD103^+ve^ T_RM_ CD8 T cells in lungs of ADJ+PLP-GLA mice were significantly (P<0.05) higher, as compared to other adjuvant groups (**Figure 5A**).

At 100 days after vaccination, ADJ+PLP-CpG and ADJ+PLP-GLA groups had significantly (P<0.05) higher frequencies and numbers of NP311-specific memory CD4 T cells in airways than other groups and both groups had significantly (P<0.05) higher numbers in airways and/or lungs than the PLP-GLA group (**Figure 5B**). Among NP311-specific CD4 T cells, the PLP-E and PLP-GLA groups had a significantly higher proportion of vascular cells and also expressed CD62L, CX3CR1 and KLRG-1 (**Figures 5C** **)**.

**Figure 6:**
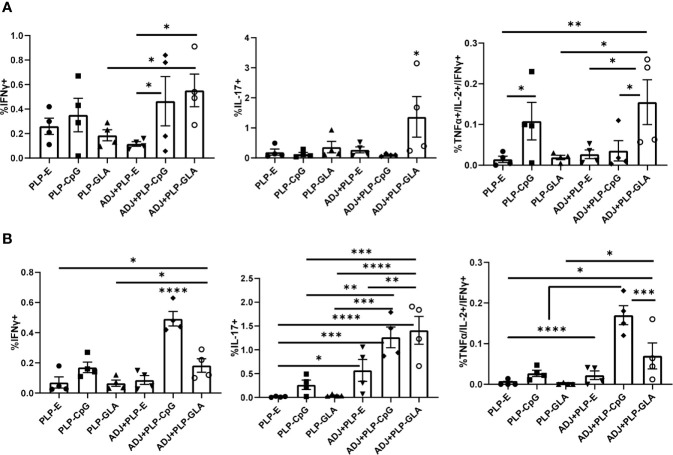
Functional polarization of memory CD8 and CD4 T cells in vaccinated mice. Cohorts of mice were vaccinated twice, as described in **Figure 2**. At 100 days after booster vaccination, lung cells were stimulated with NP366 **(A)** or NP311 **(B)** peptides. Cytokine-producing CD8 or CD4 T cells were quantified by intracellular cytokine staining. Graphs in panel A show percentages of IFN-γ- or IL-17-producing cells among CD8 T cells or TNF-α and IL-2 producing cells among IFN-γ-producing CD8 T cells. Panel B shows percentages of IFN-γ- or IL-17-producing cells among CD4 T cells or TNF-α and IL-2 producing cells among IFN-γ-producing CD4 T cells. Data are representative of three independent experiments. *, **, ***, and **** indicate significance at P < 0.1, 0.01, 0.001, and 0.0001, respectively.

The percentages of NP366-specific IFNγ+ memory CD8 T cells in lungs of ADJ+PLP-GLA or ADJ+PLP-CpG groups were higher than in ADJ+PLP-E mice. Percentages of IL-17-producing CD8 T cells in the ADJ+PLP-GLA group were significantly (P<0.05) higher than in all other groups (**Figure 6A**). While Tc17 responses in all groups contracted from D8 to D100, this contraction seemed to occur to a lesser degree in the ADJ+PLP-GLA group than the PLP-GLA group; on D8, the numbers and frequencies of NP366-specific CD8 T cells, and frequencies of Tc17 cells were not different between these two groups, but at D100, Tc17 frequencies were significantly higher in ADJ+PLP-GLA than the PLP-GLA group. Interestingly, while both ADJ+PLP-GLA or ADJ+PLP+CpG groups had strong Tc1 responses, the degree of polyfunctionality in the ADJ+PLP-GLA group was significantly higher than all other groups, except PLP-CpG. Further, ADJ appeared to reduce memory CD8 T cell polyfunctionality induced with PLP-CpG but increase polyfunctionality induced with PLP-GLA.

Lungs from all groups contained robust frequencies of IFNγ+ memory CD4 T cells at D100, however, ADJ+PLP-CpG group had significantly (P<0.05) higher frequencies of IFNγ-producing NP311-specific memory T cells, than other groups (**Figure 6B**). ADJ+PLP-CpG and ADJ+PLP-GLA were superior to PLP-CpG and PLP-GLA in inducing IL-17-producing and/or polyfunctional memory CD4 T cells (**Figure 6B**). When the functionality of both memory CD4 and CD8 cells are taken together, we observed a similar trend, which is that ADJ+PLP-GLA induced the most robust and balanced Tc1/Th1 and Th17/Tc17 responses.

### ADJ+PLP-GLA Induces Durable and Potent Immunity to Influenza Virus

**Figure 7:**
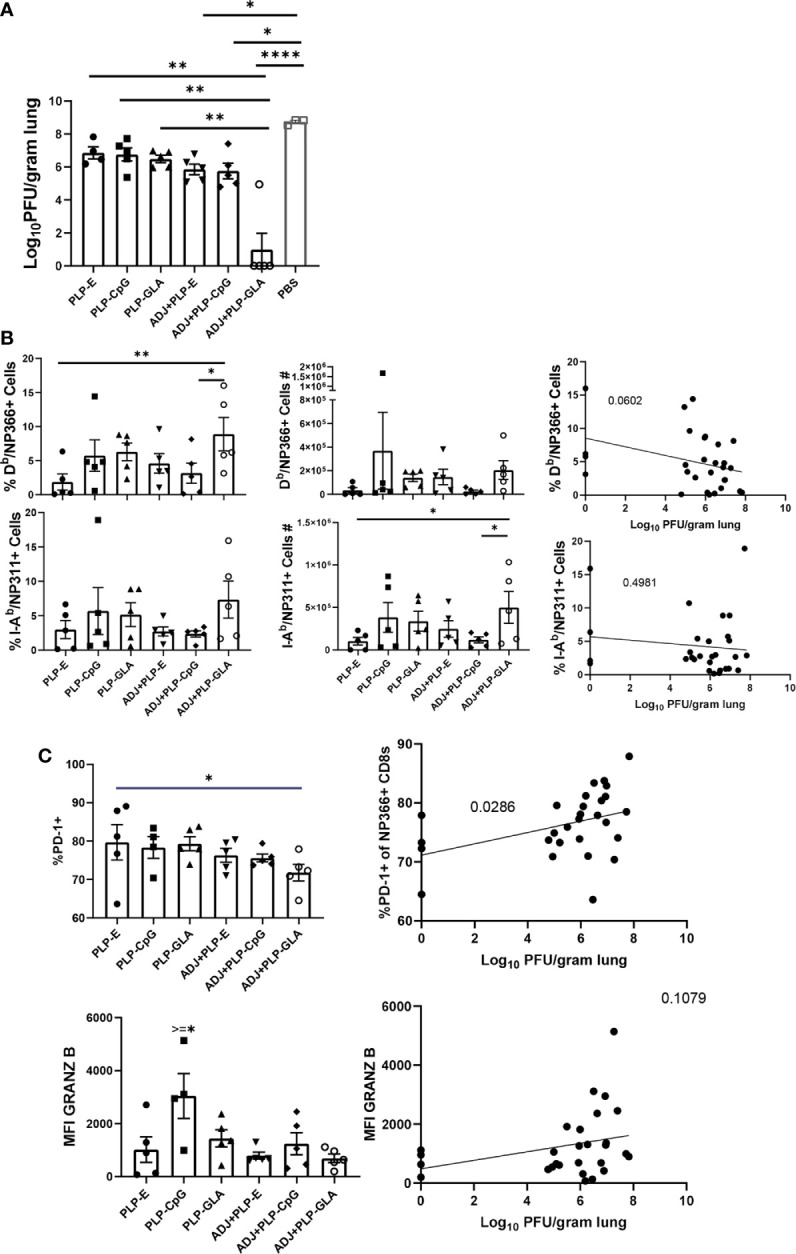
Vaccine-induced protective immunity influenza virus. Cohorts of mice were vaccinated twice, as described in **Figure 2**. At 101 days after booster vaccination, mice were challenged with H1N1/PR8 strain of influenza A virus; unvaccinated mice were challenged as controls. Viral titers were quantified in the lungs on D6 after challenge **(A)**. Percentages or numbers of NP366-specific CD8 T cells and NP311-specific CD4 T cells in lungs **(B)**. Graphs show percentages of cells among the gated NP366-specific CD8 T cells or expression levels of granzyme B in the gated NP366-specific CD8 T cells **(C)**. Data are representative of two independent experiments. *, **, ***, and **** indicate significance at P < 0.1, 0.01, 0.001, and 0.0001, respectively. Linear regression curves were plotted for data from individual mice for the indicated cell frequency plotted against its Log_10_ viral titer value.

Cohorts of mice were vaccinated twice (at an interval of 3 weeks) intranasally with NP protein formulated in various adjuvants. At day 101 after booster vaccination, vaccinated and unvaccinated animals were challenged with a lethal dose of H1N1 PR8 influenza virus. At D6 post virus challenge, we quantified recall T cell responses and viral titers in the lungs. While vaccination with all PLP formulations resulted in at least ~2 Log_10_ reduction in lung viral titers, the ADJ+PLP-GLA vaccine conferred the highest degree of viral control, reducing viral titers by nearly 7 Log_10_ PFU/gram of lung, as compared to mock (saline) vaccinated mice (**Figure 7A**). Lungs of 4/5 animals in the ADJ+ PLP-GLA group contained no detectable infectious virus, indicating nearly complete control of lung viral replication within D6 after challenge.

Interestingly, total numbers and frequencies of recall antigen-specific CD4 and CD8 T cells did not vary significantly between vaccinated groups at this time point (**Figure 7B**). However, there was a nearly significant (P<0.05) relationship between frequencies of NP366-specific CD8 T cells and viral lung titers (**Figure 7B**), whereas frequencies or numbers of CD4 T cells did not exhibit such a strong trend. Additionally, there were no noteworthy phenotypic or transcriptional differences between adjuvant groups in CD8 T cells in terms of CD49a, CD62L, CD69, CD103, CD127, CXCR3, CX3CR1, KLRG-1, TBET, EOMES, and IRF4 expression. However, we noticed a strong trend for reduced PD-1 expression on recall CD8 T cells in ADJ containing groups, particularly for ADJ+PLP-GLA, and there was a strong correlation between PD-1 expression levels and increased viral lung titers (**Figure 7C**). Further, granzyme B levels were significantly upregulated in recall CD8 T cells in the PLP-CpG group, and there was a negative trend for granzyme B levels and viral titer. Increased PD-1 and granzyme B expression in CD8 T cells might be suggestive of ongoing antigenic stimulation and cytolysis in mice, associated with delayed viral clearance. Finally, we did not observe any strong or clear trends between the aforementioned parameters on recall CD4 T cells and viral control.

### Recall T Cell Function Intimately Associates With Influenza Virus Control

**Figure 8:**
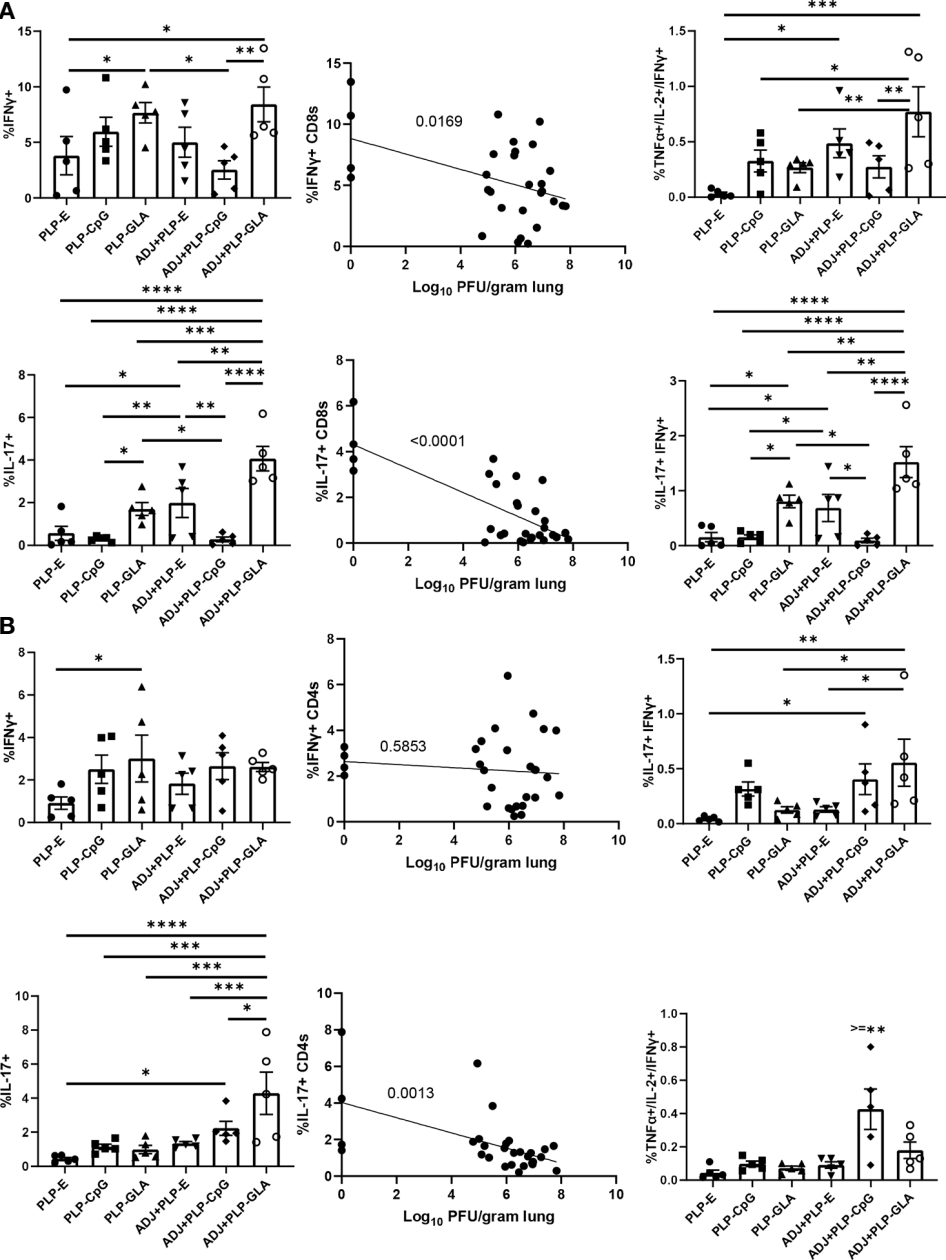
Functional polarization of recall CD8 and CD4 T cells. Cohorts of mice were vaccinated twice, as described in **Figure 2**. 101 days after booster vaccination, mice were challenged with H1N1/PR8 strain of influenza A virus. Lung cells were stimulated *ex vivo* with NP366 or NP311 peptides for 5 h. The percentages of NP366-stimulated CD8, or NP311-stimulated CD4 T cells that produced IFN-γ, IL-17, TNF-α, and IL-2 were quantified by intracellular cytokine staining. Graphs show the percentages of cytokine-producing cells among the gated CD8 T cells or TNF-α and IL-2-producing cells among gated IFN-γ-producing CD8 T cells **(A)**. Graphs show the percentages of cytokine-producing cells among CD4 T cells or TNF-α and IL-2-producing cells among gated IFN-γ-producing CD4 T cells **(B)**. Data are representative of two independent experiments. *, **, ***, and **** indicate significance at P < 0.1, 0.01, 0.001, and 0.0001, respectively. Linear regression curves were plotted for data from individual mice for the indicated cell frequency plotted against its Log_10_ viral titer value.

Following influenza virus challenge, all PLP groups showed robust IFNγ+ CD8 T-cell recall responses measured by *ex vivo* NP366 peptide stimulation (**Figure 8A**). There was a clear and strong association between the frequency of IFNγ expression in CD8 T cells and the degree of viral control in lungs (**Figure 8A**). ADJ+PLP-GLA seemed to drive increased polyfunctionality among recall CD8 T cells (**Figure 8A**). With the exception of ADJ+PLP-CpG, both ADJ and PLP-GLA groups had significantly increased frequencies of IL-17 expression during virus-induced CD8 T cell recall, with the ADJ+PLP-GLA group having significantly (P<0.05) higher levels than all other groups (**Figure 8A**). Indeed, ADJ+PLP-GLA had the greatest Tc17 recall response and the lowest lung viral titers, and overall there was an extremely close association between IL-17 expression in CD8 T cells and viral control (**Figure 8A**), such that even excluding the ADJ+PLP-GLA animals that had no detectable lung titer from the correlation analysis still resulted in a significant association (**Supplementary Figure 6**).

For CD4 Th1 recall responses, while all groups had robust IFNγ+ CD4 T-cell responses, there were no noteworthy differences between groups and there did not appear to be a strong association with viral control (**Figure 8B**). Interestingly, ADJ+PLP-CpG had significantly higher frequencies of polyfunctional CD4 T cells than all other groups. On the other hand, the ADJ+PLP-GLA group had a significantly (P<0.05) higher recall Tc17 response than other groups, and frequencies of IL-17+ cells among CD4 T cells significantly correlated with reduced viral lung titer (**Figure 8B**). Overall, the robust and balanced Tc1/Th1 and Th17/Tc17 functional responses elicited by the ADJ+PLP-GLA vaccine were associated with effective viral control.

### ADJ+PLP-GLA Induces Durable Pulmonary T-cell Immunity to Influenza Virus

**Figure 9:**
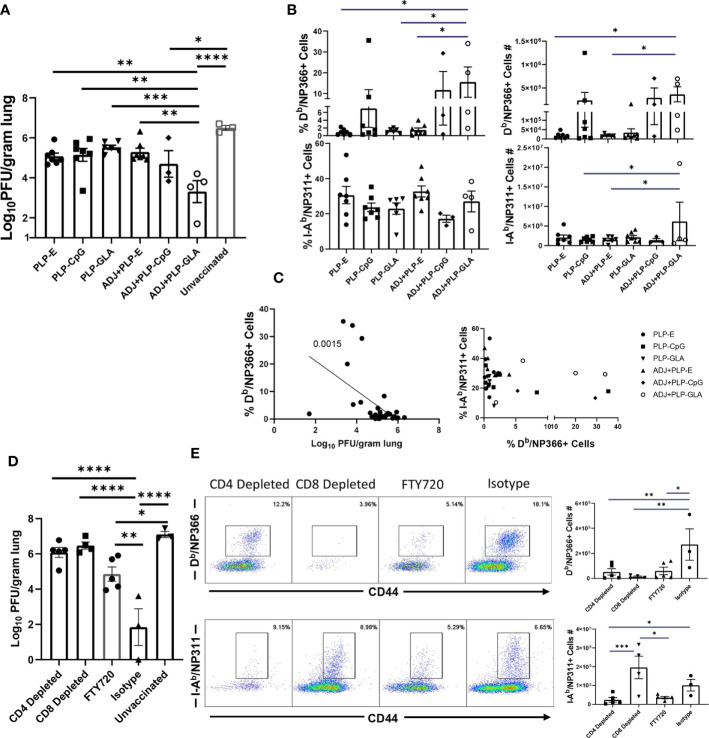
Long term memory and role of CD4 and CD8 T cells in vaccine-induced immunity to influenza virus. Three hundred sixty-two days after booster vaccination, mice were challenged with H1N1/PR8 strain of influenza A virus **(A–C)**. Viral titers were quantified in the lungs on D6 after challenge **(A)**. Numbers and frequencies of NP366-specific CD8 T cells and NP311-specific CD4 T cells in lungs **(B)**. The linear regression curve was plotted for data from individual mice frequencies of NP366-specific CD8 T cells or NP311-specific CD4 T cells plotted against its Log_10_ viral titer value, **(D, E)**: Mice were vaccinated with NP formulated in ADJ+PLP-GLA, and 3 weeks later, either CD4 or CD8 T cells were depleted immediately prior to and during a lethal H1N1 PR8 influenza challenge. An additional cohort of ADJ+PLP-GLA vaccinated mice was treated with FTY720 immediately before and during viral challenge. Viral titers were quantified in the lungs on D6 after challenge **(D)**. Numbers and frequencies of NP366-specific CD8 T cells and NP311-specific CD4 T cells in lungs **(E)**. *, **, ***, and **** indicate significance at P < 0.1, 0.01, 0.001, and 0.0001, respectively.

To test the ability of PLP based adjuvants to elicit durable immunity, cohorts of mice were vaccinated twice (3 weeks apart) with NP protein formulated in various adjuvants. Vaccinated and unvaccinated animals were challenged with a lethal dose of H1N1 PR8 influenza virus at day 362 after booster vaccination. At D6 post virus challenge, we quantified recall T cell responses and viral titers in the lungs. The ADJ+PLP-GLA vaccine conferred effective viral control at this late memory time point, reducing viral titers by nearly 3 Log_10_ PFU/gram of lung, in comparison to mock (saline) vaccinated mice (**Figure 9A**). Note that vaccination with all other PLP formulations resulted in a modest 0.5–1 Log_10_ reduction in lung viral titers. NP366-specific CD8 T-cell frequencies and total numbers were consistently higher in the ADJ+PLP-GLA group (**Figure 9B**), and majority of mice that received other vaccine formulations exhibited poor CD8 T cell recall responses. Interestingly, frequencies and total numbers of NP311-specific recall CD4 T cells were high amongst all vaccine groups (**Figure 9B**). At this late time point, reduction of viral burden in lungs closely associated with the magnitude of NP366-specific CD8 T cell recall response (**Figure 9C**). In general mice vaccinated with ADJ+PLP-GLA had high frequencies of both CD4 and CD8 T cells, as compared with other groups, which suggested that both sets of T cells may be important for viral control at this time point (**Figure 9C**).

### Both CD4 and CD8 T Cells Are Required for PLP Vaccine-Induced Protection to Influenza Virus Challenge

As detailed above, ADJ+PLP-GLA elicited potent NP-specific CD4 and CD8 T cell responses during effector and memory time points, which was associated with influenza virus control. To determine the relative roles of T cell subsets in protective immunity, we vaccinated mice with NP protein formulated in ADJ+PLP-GLA. Three weeks after vaccination, we depleted CD4 or CD8 T cells, immediately prior to challenge with a lethal dose of H1N1 PR8 influenza A virus. Depletion of CD4 and CD8 T cells resulted in selective loss of NP311-specific CD4 T cells and NP366-specific CD8 T cells, respectively in lungs (**Figure 9E**). Further, CD4 T-cell depletion significantly reduced CD8 T-cell recall, which suggested that CD4 T cells support recall responses of CD8 T cells. CD8 T cell depletion appeared to increase CD4 T cell recall (**Figure 8E**), which might be suggestive of compensatory CD4 T cell responses, in the absence of CD8 T cells. Vaccinated mice treated with isotype control antibodies displayed >4 Log_10_ reduction in viral load in lungs, as compared to unvaccinated controls (**Figure 9D**). Remarkably, antibody-induced depletion of CD4 or CD8 T cell subsets resulted in significantly (P<0.05) increased viral burden in lungs, as compared to isotype control antibody treatment (**Figure 9D**). These findings suggested that both CD4 and CD8 T cells play important roles in controlling influenza viral burdens in lungs of vaccinated mice.

To further investigate the importance of lung T_RM_ in protection against influenza virus challenge, a cohort of ADJ+PLP-GLA-vaccinated mice was treated with FTY720 (Fingolimod), a functional antagonist of sphingosine-1-phosphate receptor (S1PR), that prevents lymphocyte egress from secondary lymphoid organs. As expected, FTY720 treatment markedly reduced the numbers of circulating CD4 and CD8 T cells (**Supplementary Figure 7**). Viral burden in lungs of FTY720-treated animals was significantly lower than in unvaccinated controls (~ 2 Log_10_ reduction). However, lung viral load in FTY720-treated animals was significantly (P<0.05) higher than in lungs of mice that were not treated with FTY720 (Isotype control antibodies). Taken together, these data suggested that FTY720 treatment compromised memory CD4 and CD8 T-cell-mediated influenza viral control in vaccinated mice. This in turn implied that both T_RM_ and egress of lymphoid memory T cells might be important for influenza virus control in vaccinated mice. This interpretation comes with a caveat, because FTY720 has been reported to inhibit activation and effector function of T cells (29, 30) and this in turn might have affected recall responses of memory T cells including T_RM_, leading to ineffective viral control in lungs.

## Discussion

Programming potent and durable B and T cell memory is the goal of vaccination programs. Live viral vaccines such as the Yellow fever vaccine (YFV) and the small pox vaccine engender immunity that lasts for decades after vaccination (31). Studies of YFV suggest that engagement of multiple innate immune receptors early in the infection may be key to the programming of long-lived immunological memory (32). This paradigm has triggered a multitude of investigations to explore the possibility of using TLR agonists as adjuvants to program durable immunological memory. One of the downsides of using TLR agonists, such as CpG, in soluble form is their diffusion from vaccination site, leading to systemic toxicity in vaccinees (33). To circumvent this problem and mimic the biophysical attributes of pathogens and their interactions with pattern recognition receptors, we engineered biodegradable PLGA microparticles (i.e. PLPs) that were loaded with optimized densities of TLR agonists CpG and GLA (19). In this manuscript, we document that mucosal delivery of CpG- or GLA-loaded PLPs elicit unexpectedly potent mucosally imprinted antigen-specific CD4 and CD8 T cell responses in the respiratory tract. Interestingly, we find that PLP-CpG and PLP-GLA stimulate disparate transcriptional programs that evoke distinct phenotypic and functional differentiation of antigen-specific T cells. Further, we show that the combination of PLP-GLA, but not PLP-CpG, with the nanoemulsion adjuvant ADJ synergistically augmented the magnitude of lung-resident T cell memory and protective immunity to a lethal influenza virus infection. These studies highlight how the mode of TLR agonist presentation can be leveraged to achieve enhanced immunogenicity without toxicity, and how this feature can be combined with the antigen presenting properties of a nano-emulsion adjuvant to program effective T cell-based protective immunity in the respiratory tract.

*In vitro* studies show that PLP-CpG, but not PLP-GLA, triggers IFNβ production by BMDCs. Conversely, only PLP-GLA, but not CpG, stimulate IL-1β production when combined with ADJ; differential abilities of PLP-CpG and PLP-GLA in eliciting IFNβ versus IL-1β production need further investigation, but TLR4 agonists are known to promote inflammasome activation and IL-1β release (34, 35). Despite this difference in IFNβ and IL-1β induction, both PLP-CpG and PLP-GLA elicited strong, yet comparable levels of effector CD8 and CD4 T cell responses *in vivo*. This data suggests that: 1) lack of IFNβ induction *in vitro* does not predict failure by an adjuvant to induce T cell responses *in vivo*; 2) PLP-CpG and PLP-GLA likely stimulate different arrays of cytokines in DCs *in vivo*; 3) PLP-CpG and PLP-GLA might engage different pathways to stimulate T cell activation and expansion in the respiratory tract. Consistent with this idea, it is noteworthy that PLP-CpG and PLP-GLA differ when the differentiation of T cells are compared. In comparison to PLP-GLA, PLP-CpG tended to drive T cells towards terminal differentiation, based on elevated expressions of KLRG-1, CX3CR1, TBET, and EOMES. CpG is known to induce high levels of IL-12 (36), and it is likely that higher induction of IL-12 in DCs by PLP-CpG underlies greater levels of TBET and terminal differentiation of effector T cells in PLP-CpG-vaccinated mice. Pertaining to functional polarization of T cells, PLP-CpG elicited primarily Tc1 CD8 T cells and Th1 CD4 T cells secreting IFNγ, but only a fraction of CD4 T cells also secreted IL-17α. In contrast, PLP-GLA promotes functionally broad CD8 and CD4 T cell responses that secreted IFNγ and/or IL-17α. The differential functional polarization of T cells in PLP-CpG and PLP-GLA-vaccinated mice might be linked to disparate levels of IL-12 and inflammasome activation, respectively (19, 36, 37). In addition to the inflammatory milieu, the strength and/or duration of TCR signaling affects the differentiation of T cells (38). In this context, we have previously reported that ADJ in combination with soluble CpG enhance TCR signaling and promote terminal differentiation of effector CD8 and CD4 T cells (17). By contrast, ADJ in combination with soluble GLA results in dampened TCR signaling, leading to limited terminal differentiation of effectors and development of a larger pool of memory T cells (17). Since TLR4 engagement is known to downregulate antigen-triggered TCR signaling (39), we theorize that disparate TCR signaling induced by CpG and GLA might underlie differential T cell responses to ADJ+CpG versus ADJ+GLA in vaccinated mice.

Total numbers of influenza-specific lung T_RM_ CD8 T cells correlate with protection from rechallenge (10–12). We have previously reported that ADJ, a carbomer-containing nanoemulsion adjuvant induces T_RM_s in lungs and protects against pathogenic influenza A virus infection (16). In the current study, we explored whether combining ADJ with PLP-CpG or PLP-GLA augmented the adjuvanticity of ADJ and increased the numbers of lung T_RM_ CD8 T cells. PLP-CpG, PLP-GLA, and ADJ alone did not significantly differ in terms of the numbers of lung T_RM_ CD8 T cells. Although ADJ suppresses PLP-CpG-induced IFNβ production *in vitro*, ADJ did not adversely affect CD8 or CD4 T cell responses to PLP-CpG. The numbers of effector CD8 T cells (at the peak of the response) or memory CD8 T cells were not significantly different in PLP-CpG versus ADJ+PLP-CpG groups, though as expected, there was contraction of NP366+ CD8 T cells in lungs from D8 to D100 post boost. Thus, combining ADJ with PLP-CpG did not alter the development of memory CD8 T cells. In striking contrast, combining ADJ with PLP-GLA generally enhanced the number of effector and memory CD8/CD4 T cells, as compared to ADJ or PLP-GLA alone; ADJ+PLP-GLA induced the largest cohort of lung- and airway-resident T_RM_s, as compared to all other groups. This in turn correlated strongly with the most effective protective immunity against pathogenic influenza virus infection. As discussed above, PLP-CpG with or without ADJ promotes high level of expression of TBET leading to a greater degree of differentiation of KLRG-1^HI^/CX3CR1^HI^ CD8 T cells. By contrast, the combination of ADJ+PLP-GLA maximizes effector/memory mucosal NP366-specific CD8 T cells and T_RM_ frequencies, and downregulates the levels of KLRG-1, CX3CR1, Granzyme B, TBET, EOMES, and Ki67 (40, 41). Furthermore, engaging the RORγ/Tc17/Th17 differentiation program by ADJ+PLP-GLA might enhance differentiation of long-lived stem cell-like memory T cells (42). Nonetheless, our results are consistent with the axiom that T_RM_ arise from less differentiated effector cells, and terminally differentiated effector cells have diminished capacity to develop into T_RM_s (43, 44). Collectively, our findings support the rationale and feasibility of combining adjuvants to mitigate terminal differentiation of effectors and enhance the development of T_RM_.

Memory T cell-dependent protection against influenza virus is dictated by the number of memory CD8 T cells in airways and lung parenchyma (10–12, 45). Consistent with these reports, we find that ADJ+PLP-GLA mice contained significantly greater numbers of T_RM_ and memory T cells in airways, which correlated strongly with the most effective protection against pathogenic influenza infection. Additionally, we find a strong correlative link between recall CD4/CD8 T cell functionality and reduction of influenza virus lung titers, specifically in Tc1/Tc17 and Th1/Th17 responses (factors associated with protection to influenza in the ADJ+PLP-GLA group are listed in **Supplementary Table 1**). The role of IFNγ, and its production in recall lung CD8 T cell responses is established in controlling influenza viral lung replication (46–49). While we also observed a strong correlation between IFNγ expression in lung CD8 T cells and reduction in viral titers, this association did not apply for lung CD4 T cells, indicating that perhaps Tc1 cells are more important than Th1 cells in controlling influenza virus. Interestingly, production of IL-17α from both CD8 and CD4 T cells was closely associated with reduction in lung viral lung titers in this study. IL-17α-producing T cells are well known to be involved in fungal immunity (50, 51), however the role of Tc17/Th17 responses in control of influenza infection is only recently emerging. Lung Tc17 cells appear to be a distinct subset of CD8 T cells, compared with Tc1 cells, and are associated with protection from rechallenge (52, 53). Influenza virus-specific lung Th17 have also been implicated in protection from influenza (54, 55). Overall, more mechanistic studies are required to carefully dissect and correlate the roles of individual functional responses in vaccine-induced lung T cells to influenza viral control.

While antibody-mediated protection against influenza virus is type and subtype specific, memory T cells that recognize conserved epitopes in the internal proteins, such as nucleoprotein, provide heterosubtypic immunity to influenza A virus (56, 57). Hence, there is an impetus to identify strategies to elicit T cell immunity in the lungs towards an universal influenza vaccine (56). Our studies show that both CD4 and CD8 T cells are essential for mediating vaccine immunity to influenza A virus, but the exact mechanisms remain unknown. Our data strongly suggest T-cell-dependent immunity, but our studies do not exclude a role for antibodies in vaccine-induced protection. It is less likely that NP protein induces virus neutralizing antibody responses, but we cannot exclude the possibility that NP-specific antibodies might promote antigen uptake by FcR-dependent mechanisms, leading to enhanced antigen presentation to CD8 and CD4 T cell responses during vaccination (58–60).

It is clear that recovery from influenza virus infection leads to memory T cell-dependent immunity to reinfection, but this immunity is not durable (10–12). Protection afforded by ADJ+PLP-GLA vaccination was evident at both 100 and 362 days post boost, and T cell dependant. In comparison to published work, we find that T cell responses elicited by ADJ+PLP-GLA differ from those induced by influenza virus infection in two key aspects: 1) A larger proportion of NP366-specific memory CD8 T cells elicited by ADJ+PLP-GLA are mucosally imprinted (CD69+/CD103+) and reside in the lung parenchyma at memory (D100) measurements (**Figure 5A**), while majority of influenza virus-elicited NP366 specific memory CD8 T cells at comparable time points are CD69-/CD103- and/or reside in the vascular compartment (61, 62); 2) Vaccination with PLP adjuvants, particularly ADJ+PLP-GLA, elicits durable antigen specific CD4 memory (**Figure 5B**), unlike influenza infection (63). Mechanisms underpinning differences in vaccine- versus infection-induced T-cell memory needs further investigation.

There are no FDA-approved mucosal vaccines formulated in adjuvants that are known to elicit protective T cell immunity in the respiratory mucosa. In this manuscript, we have explored novel ways of presenting immune components to the immune system to maximize antigen presentation to T cells and evoke innate immune responses that program a strong and enduring mucosal T cell response in the respiratory tract. Specifically, we have identified a novel vaccine formulation consisting of influenza virus NP, TLR-loaded PLPs, and a nanoemulsion adjuvant, that elicits robust mucosally-imprinted T cell memory in the respiratory tract and affords effective protective immunity to a pathogenic influenza virus infection in mice. These findings have significant implications in the development of T cell-based vaccines against respiratory viral pathogens such as influenza virus and SARS-CoV-2.

## Data Availability Statement

The raw data supporting the conclusions of this article will be made available by the authors, without undue reservation.

## Ethics Statement

The animal study was reviewed and approved by Animal Care and Use Committee at the UW School of Veterinary Medicine.

## Author Contributions

MS conceived experiments, analyzed data, and edited the paper. BK-B conceived experiments, performed experiments, analyzed data, and wrote the paper. RT performed experiments, analyzed data, and edited manuscript. WL, PP, GV, CM, DG, TL, BP and AL performed experiments. YK provided critical reagents. All authors contributed to the article and approved the submitted version.

## Funding

This study was supported by PHS grants from the National Institutes of Health (grant# U01AI124299 and R21 AI149793 ) and funds from the John E. Butler Professorship to MS. WL was supported by a pre-doctoral fellowship from the American Heart Association. KR was supported by a PHS grant from the National institutes of Health (grant #:U01AI124270).

## Conflict of Interest

The authors declare that the research was conducted in the absence of any commercial or financial relationships that could be construed as a potential conflict of interest.
