# Supplementary material for: Polymeric Pathogen-Like Particles-Based Combination Adjuvants Elicit Potent Mucosal T Cell Immunity to Influenza A Virus
Source: Front Immunol. 2021 Mar 4;11:559382. doi: 10.3389/fimmu.2020.559382 (PMC7986715; doi:10.3389/fimmu.2020.559382)
Supplement: Supplementary file 1 [file DataSheet_1.pdf]

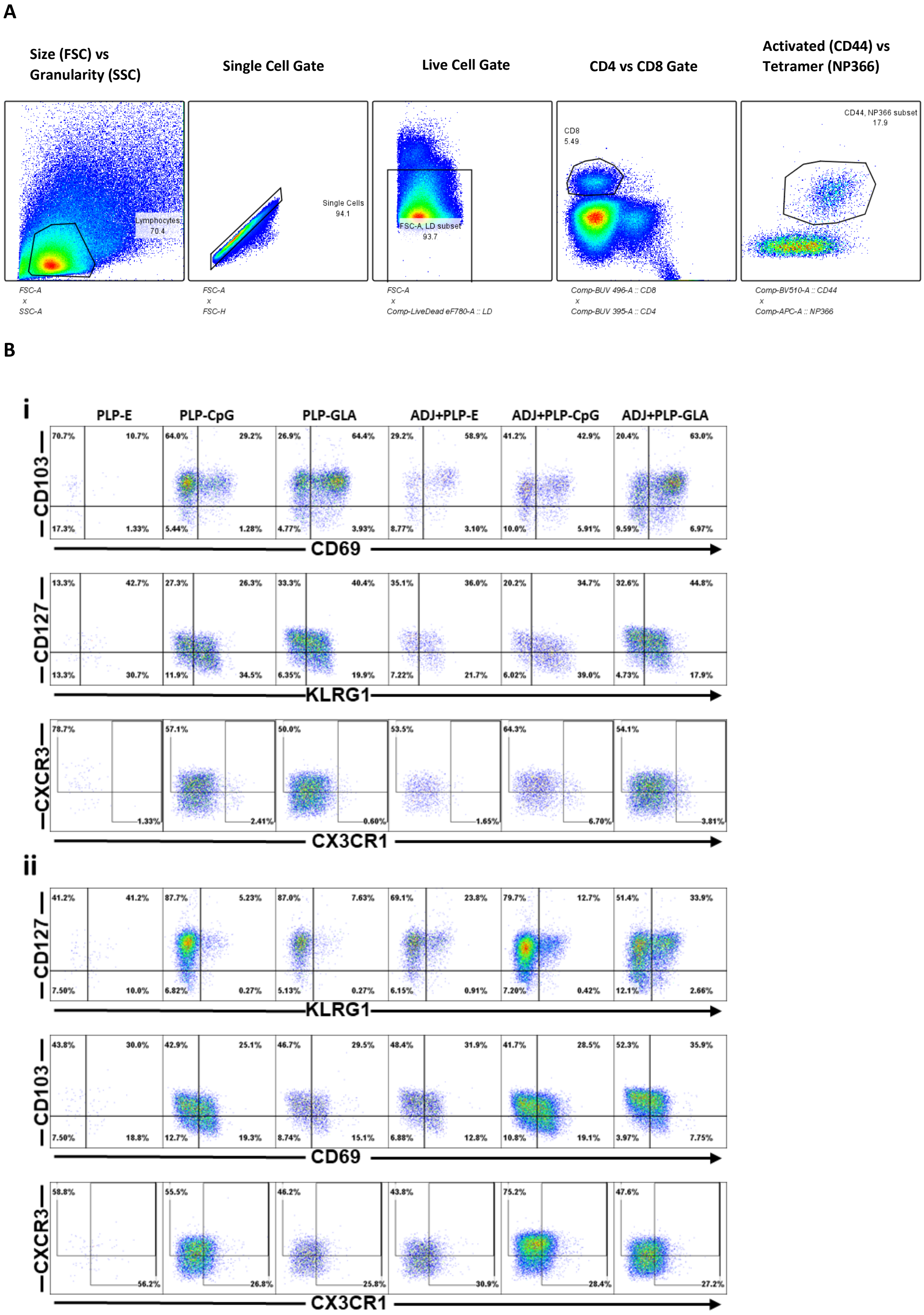
A
Size (FSC) vs Granularity (SSC)
Single Cell Gate
Live Cell Gate
CD4 vs CD8 Gate
Activated (CD44) vs Tetramer (NP366)
Lymphocytes 70.4
Single Cells 94.1
FSC-A, LD subset 93.7
CD8 5.49
CD44, NP366 subset 17.9
FSC-A x SSC-A
FSC-A x FSC-H
FSC-A x Comp-LiveDead eF780-A :: LD
Comp-BUV 496-A :: CD8 x Comp-BUV 395-A :: CD4
Comp-BV510-A :: CD44 x Comp-APC-A :: NP366
B
i
PLP-E
PLP-CpG
PLP-GLA
ADJ+PLP-E
ADJ+PLP-CpG
ADJ+PLP-GLA
CD103
CD69
70.7% 10.7% 17.3% 1.33%
64.0% 29.2% 5.44% 1.28%
26.9% 64.4% 4.77% 3.93%
29.2% 58.9% 8.77% 3.10%
41.2% 42.9% 10.0% 5.91%
20.4% 63.0% 9.59% 6.97%
CD127
KLRG1
13.3% 42.7% 13.3% 30.7%
27.3% 26.3% 11.9% 34.5%
33.3% 40.4% 6.35% 19.9%
35.1% 36.0% 7.22% 21.7%
20.2% 34.7% 6.02% 39.0%
32.6% 44.8% 4.73% 17.9%
CXCR3
CX3CR1
78.7% 1.33%
57.1% 2.41%
50.0% 0.60%
53.5% 1.65%
64.3% 6.70%
54.1% 3.81%
ii
CD127
KLRG1
41.2% 41.2% 7.50% 10.0%
87.7% 5.23% 6.82% 0.27%
87.0% 7.63% 5.13% 0.27%
69.1% 23.8% 6.15% 0.91%
79.7% 12.7% 7.20% 0.42%
51.4% 33.9% 12.1% 2.66%
CD103
CD69
43.8% 30.0% 7.50% 18.8%
42.9% 25.1% 12.7% 19.3%
46.7% 29.5% 8.74% 15.1%
48.4% 31.9% 6.88% 12.8%
41.7% 28.5% 10.8% 19.1%
52.3% 35.9% 3.97% 7.75%
CXCR3
CX3CR1
58.8% 56.2%
55.5% 26.8%
46.2% 25.8%
43.8% 30.9%
75.2% 28.4%
47.6% 27.2%

Supplementary Figure 1. Gating strategy for visualization and analysis of antigen-specific T cells. A) For all antigen specific cells, the general gating strategy is shown: FSC vs. SSC mononuclear cell gate -> single cell gate -> live cell gate -> CD4 or CD8 cells -> gated antigen-specific cells for further characterization. B) FACs plots are shown for selected phenotypic analysis of NP366-specific CD8 T cells (i) or NP311-specific CD4 T cells (ii) isolated from mouse lungs D8 post boost. FACS plots in B are gated on D^b/NP366 tetramer-binding CD8 T cells (i) or I-A^b/NP311 tetramer-binding CD4 T cells.

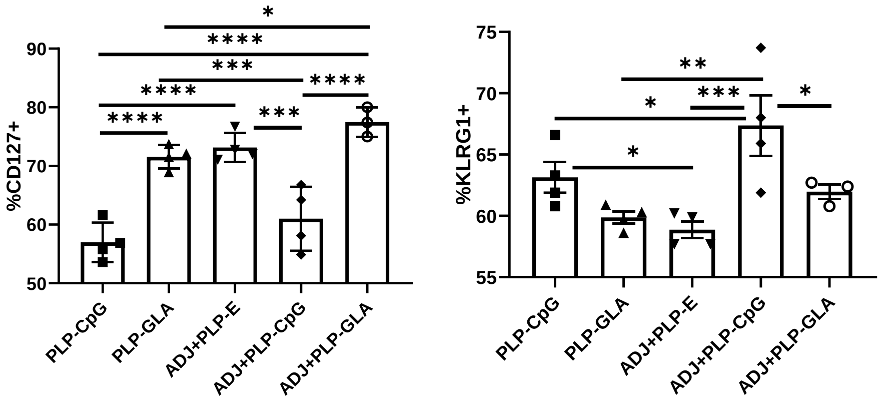

Supplementary Figure 2: CD127 and KLRG1 expression on effector CD8 T cells in response to adjuvanted vaccines. At day 8 post booster vaccination, cells from lungs were stained with D^b/NP366 tetramers, anti-CD8, anti-CD127 and anti-KLRG-1. Frequencies of CD127+ and KLRG1+ cells among NP366-specific CD8 T cells were compared between different adjuvants.

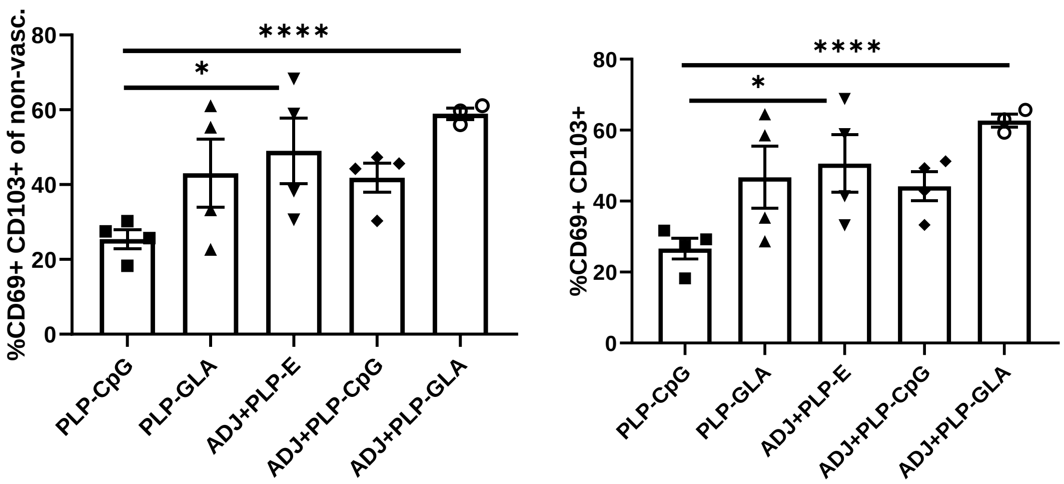

Supplementary Figure 3. Frequencies of CD69+/CD103+ CD8 T cells among non-vascular and total NP366-specific CD8 T cells. At day 8 post booster vaccination, cells from lungs were stained with D^b/NP366 tetramers, anti-CD8, anti-CD69 and anti-CD103. Frequencies of CD69+ and CD103+ cells among non-vascular and total NP366-specific CD8 T cells were determined by flow cytometry.

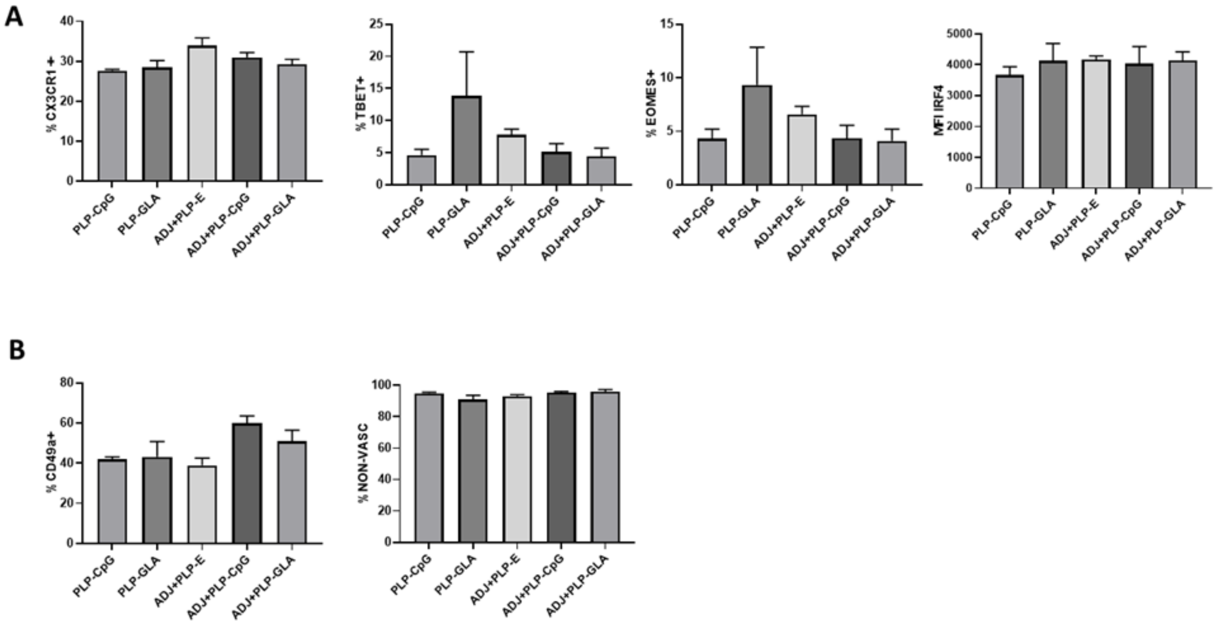

Supplementary Figure 4: Pulmonary effector CD4 T cell responses to adjuvanted vaccines. At day 8 post booster vaccination, mice were administered IV with anti-CD45.2 antibodies prior to euthanasia (to stain vascular cells). Cells from lungs were stained with I-A^b/NP311 tetramers and the indicated antibodies. (A) and (B) plots show percentages of the CD49a+ or vascular cells among the gated tetramer-binding CD4 T cells. Data are representative of three independent experiments.

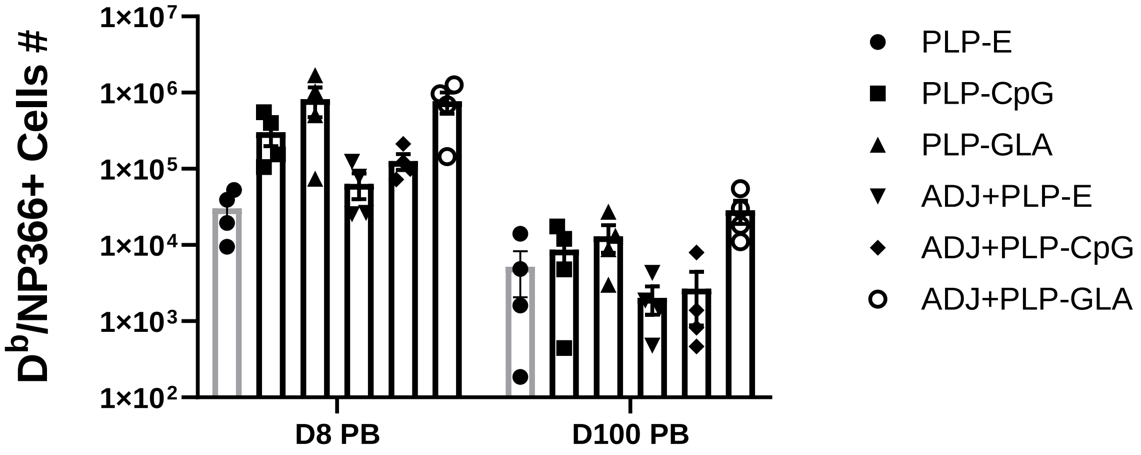

Supplementary Figure 5: Comparison of NP366+ CD8 T cell numbers in lungs detected by tetramer staining at D8 vs. D100 post boost (PB). B6 mice (n=4 all groups) were vaccinated intranasally twice with influenza virus nucleoprotein (NP) formulated with the indicated adjuvants. At day 8 and day 100 post boost, cells in the lungs were stained with ant-CD8 and D^b/NP366 tetramers, and tetramer-binding CD8 T cells were quantified by flow cytometry.

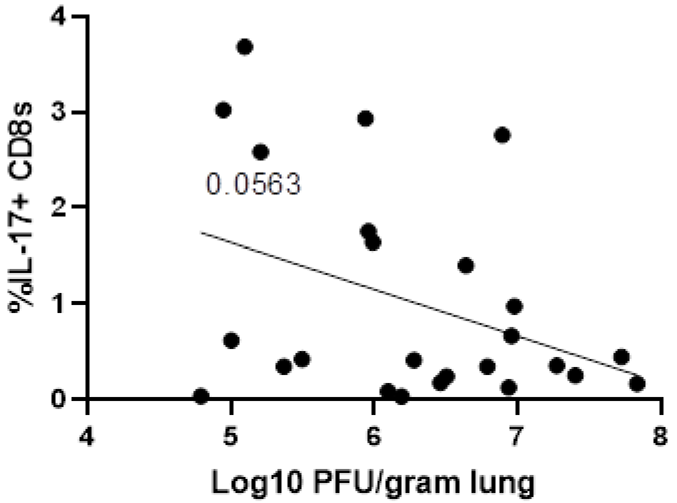

Supplementary Figure 6. Association of viral control with IL-17 production by CD8 T cells. At 101 day after booster vaccination, mice were challenged with PR8/H1N1 influenza A virus. At day 6 after virus challenge, lung viral titers and IL-17 production by NP366 peptide-stimulated CD8 T cells were quantified. Linear regression curve was plotted for data from individual mice for IL-17+ CD8 cell frequency against its Log_{10} viral titer value; mice that have no detectable lung titer were excluded from analysis.

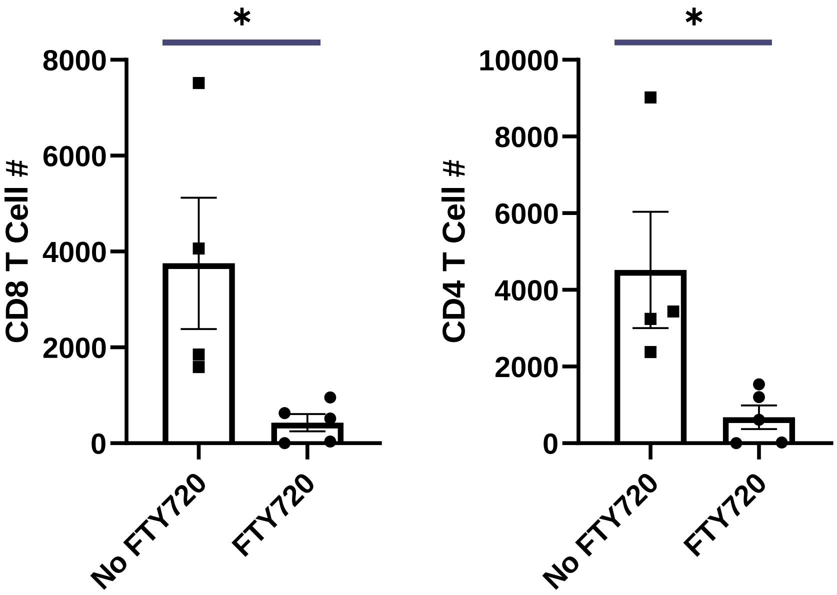

Supplementary Figure 7: CD4 and CD8 T cell counts in <u>blood</u> of FTY720-treated mice. Mice were vaccinated with NP formulated in ADJ+PLP-GLA, and three weeks later, a cohort of ADJ+PLP-GLA vaccinated mice was treated with FTY720 immediately before and during viral challenge. On day D6 post challenge, mononuclear cells isolated from 250ul of blood was stained with anti-CD4 or anti-CD8 antibodies. Total CD4 and CD8 counts are shown.

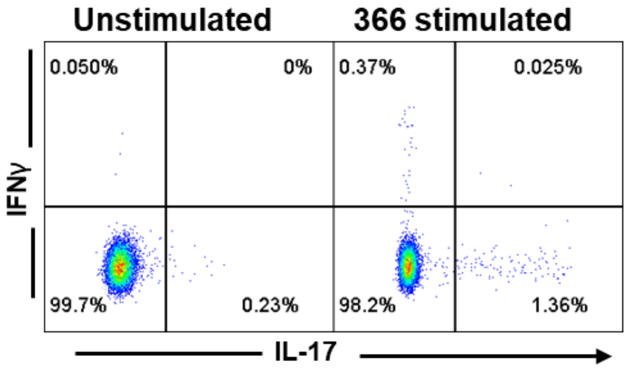

Supplementary Figure 8: Comparison of NP366 peptide stimulated and unstimulated memory CD8 T cells. On the 100th day after booster vaccination, lung cells were isolated from mice vaccinated with ADJ+PLP-GLA and stimulated *ex vivo* with NP366 peptides or media (unstimulated) for 5 hrs. The percentages of CD8 T cells that produced IFN-γ or IL-17 are shown for a concatenated pool of equally down sampled (n=4) samples.
