# Supplementary material for: Polymeric Pathogen-Like Particles-Based Combination Adjuvants Elicit Potent Mucosal T Cell Immunity to Influenza A Virus
Source: Front Immunol. 2021 Mar 4;11:559382. doi: 10.3389/fimmu.2020.559382 (PMC7986715; doi:10.3389/fimmu.2020.559382)
Supplement: Supplementary file 2 [file Table_1.pdf]

Table 1. Immunological Parameters Associated with Enhanced Protective Immunity to Influenza A Virus in ADJ+PLP-GLA group

	D8 Effector	D100 Memory	D101 Challenge Recall
**D^b/NP366+ CD8 T Cells #**	↑	↑	↑
**I-A^b/NP311+ CD4 T Cells #**	↑	↑	↑
**%IFNγ+ CD8 T Cells**	↑	↑	↑
**%IL-17α+ CD8 T Cells**	↑	↑	↑
**% IL-17α+ CD4 T Cells**	↑	↑	↑
**PD-1 Expression on NP366-Specific CD8 T Cells**	↓	NA	↓
**Granzyme B expression by NP366-Specific CD8 T Cells**	↓	NA	↓
**Mucosally Imprinted Effector or Tissue-Resident Memory CD8 T Cells**	↑	↑	NA

NA: Not Applicable
