# Supplementary material for: Polymeric Pathogen-Like Particles-Based Combination Adjuvants Elicit Potent Mucosal T Cell Immunity to Influenza A Virus
Source: Front Immunol. 2021 Mar 4;11:559382. doi: 10.3389/fimmu.2020.559382 (PMC7986715; doi:10.3389/fimmu.2020.559382)
Supplement: Supplementary file 3 [file Table_2.pdf]

REAGENT or RESOURCE	SOURCE	IDENTIFIER
Antibodies		
Rat Anti-CD4-BUV496-conjugated (GK1.5)	BD Pharmingen	564667
Rat Anti-CD8-BUV395-conjugated (53-6.7)	BD Pharmingen	563786
Rat Anti-CD44-BV510-conjugated (IM7)	BD Pharmingen	563114
Hamster Anti-CD49a-BV605-conjugated (Ha31/8)	BD Pharmingen	740375
Hamster Anti-KLRG1-BV711-conjugated (2F1)	BD Pharmingen	564014
Rat Anti-CD62L-Alexa 700-conjugated (MEL-14)	BD Pharmingen	560517
Rat Anti-IL-2-PE-CF594-conjugated (JES6-5H4)	BD Pharmingen	562483
Rat Anti-TNF-BV 421-conjugated (MP6-XT22)	BD Pharmingen	563387
Rat Anti-IFN-γ-APC-conjugated (XMG 1.2)	BD Pharmingen	554413
Hamster Anti-CD279 (PD-1)-BV 650-conjugated (J43)	BD Pharmingen	744546
Hamster Anti-Mouse CD69-PE-Cy7-conjugated (H1.2F3)	BD Pharmingen	552879
Hamster Anti- CD103 Antibody-FITC-conjugated (2E7)	eBioscience	11-1031-85

Mouse anti-CD45.2 Monoclonal Antibody (104)	eBioscience	12-0454-82
Rat InVivoMAb anti-mouse CD4	BioXcell	BE0003-1
Rat InVivoMAb anti-mouse CD8α (53-6.7)	BioXcell	BE0004-1
Rat anti-EOMES-PE-eFluor 610-conjugated (Dan11mag)	eBioscience	61-4875-82
Mouse anti-T-Bet-PerCP-Cy5.5-conjugated (4B10)	eBioscience	45-5825-82
Rat anti-IRF4-FITC-conjugated (3E4)	eBioscience	11-9858-82
Mouse anti-Granzyme B-PE-conjugated (GB12)	Thermo Fisher Scientific	MHGB04
Rat anti-CD127-PerCP-Cy5.5-conjugated (A7R34)	Biolegend	135022
Rat anti-CD127-BV650-conjugated (A7R34)	Biolegend	135043
Mouse anti-CX3CR1-BV785-conjugated (SA011F11)	Biolegend	SA011F11
Hamster anti-CXCR3-BV650-conjugated (CXCR3-173)	Biolegend	126531
Rat anti-IL-17A-FITC conjugated (TC11-18H10.1)	Biolegend	506908

BV421-conjugated I-Ab tetramers bearing the NP peptide NP311 (QVYSLIRPNENPAHK)	NIH Tetramer Core Facility at Emory University	N/A
APC-conjugated-H2-Kb tetramers bearing the NP peptide NP366 (ASNENMDTM)	NIH Tetramer Core Facility at Emory University	N/A
